# Scaffold-Guided Crystallization of Oriented α-FAPbI_3_ Nanowire Arrays for Solar Cells

**DOI:** 10.1021/acsami.3c09434

**Published:** 2023-11-21

**Authors:** Aida Alaei, Seyed Sepehr Mohajerani, Ben Schmelmer, Thiago I. Rubio, Justin Bendesky, Min-Woo Kim, Yichen Ma, Sehee Jeong, Qintian Zhou, Mia Klopfenstein, Claudia E. Avalos, Stefan Strauf, Stephanie S. Lee

**Affiliations:** † Department of Chemistry and Molecular Design Institute, New York University, New York, New York 10003, United States; ‡ Department of Physics, Stevens Institute of Technology, Hoboken, New Jersey 07030, United States

**Keywords:** perovskite, solar cell, nanowire, crystal orientation, nanoconfinement

## Abstract

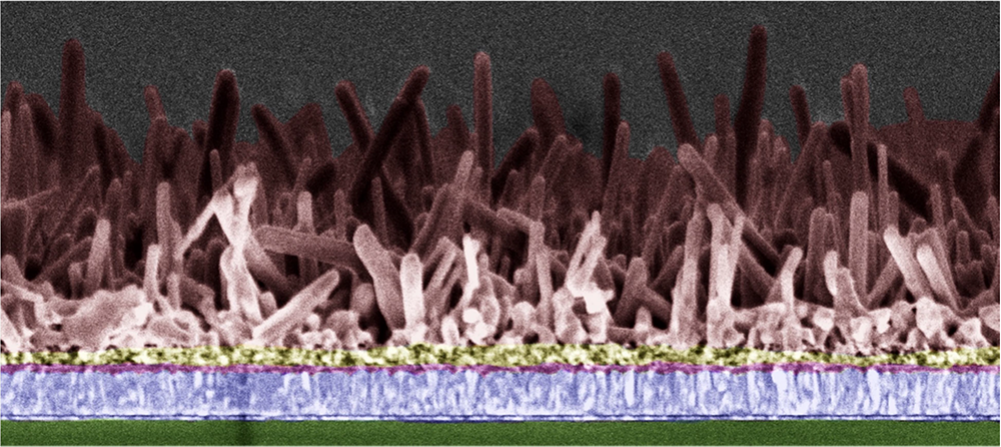

Perovskite nanowire arrays with large surface areas for efficient charge transfer and continuous highly crystalline domains for efficient charge transport exhibit ideal morphologies for solar-cell active layers. Here, we introduce a room temperature two-step method to grow dense, vertical nanowire arrays of formamidinium lead iodide (FAPbI_3_). PbI_2_ nanocrystals embedded in the cylindrical nanopores of anodized titanium dioxide scaffolds were converted to FAPbI_3_ by immersion in a FAI solution for a period of 0.5–30 min. During immersion, FAPbI_3_ crystals grew vertically from the scaffold surface as nanowires with diameters and densities determined by the underlying scaffold. The presence of butylammonium cations during nanowire growth stabilized the active α polymorph of FAPbI_3_, precluding the need for a thermal annealing step. Solar cells comprising α-FAPbI_3_ nanowire arrays exhibited maximum solar conversion efficiencies of >14%. Short-circuit current densities of 22–23 mA cm^–2^ were achieved, on par with those recorded for the best-performing FAPbI_3_ solar cells reported to date. Such large photocurrents are attributed to the single-crystalline, low-defect nature of the nanowires and increased interfacial area for photogenerated charge transfer compared with thin films.

## Introduction

Metal halide perovskites (MHPs) with the general formula of AMX_3_, in which A is an organic or inorganic cation (methylammonium, MA^+^, formamidinium, FA^+^, or Cs^+^), M is a metal cation (Pb^2+^ or Sn^2+^), and X is a halide anion (Cl^–^, Br^–^, or I^–^), are frontrunners among solution-processable materials for lightweight, large-area, and flexible optoelectronics.^1^ Of all of the possible compositions, FAPbI_3_ exhibits the smallest bandgap of 1.43 eV,^2^ resulting in broader light absorption across the solar spectrum compared to other MHPs. In recent years, FAPbI_3_ solar cells have achieved solar conversion efficiencies as high as 22%^3,4^ but typically suffer from rapid degradation.^5^ The thermodynamically stable phase of FAPbI_3_ at room temperature is the δ-phase, an insulating, nonperovskite polymorph. The optoelectronically active phase of FAPbI_3_, termed the α-phase, is typically stable only above 420 K.^6^

Confining crystals within nanoporous scaffolds is a promising strategy to stabilize metastable polymorphs and has been demonstrated in a wide range of compounds, including anthranilic acid^7^ and acetaminophen.^8^ MHPs also exhibit improved polymorph stability when confined within the 20–250 nm-diameter cylindrical nanopores of anodized aluminum oxide (AAO). MAPbI_3_, for example, exhibits decreased polymorph transition temperatures of 170 K (compared to 327 K in the bulk)^9^ and 80 K (compared to 160 K in the bulk)^10^ for the cubic-to-tetragonal and tetragonal-to-orthorhombic phases, respectively.^11^ For CsPbI_3_, the γ–δ transition is completely suppressed within both AAO^12^ and TiO_2_^13^ nanoporous scaffolds. These shifts in polymorph transition temperatures can be attributed to changes in the relative Gibbs free energies of the different polymorphs upon crystal size reduction.^13−15^ Shifting the relative polymorph stabilities in this manner can lower the processing temperatures during device fabrication.

While nanoconfinement of MHPs in scaffolds can dramatically improve the stability of the desired polymorphs, incorporating these scaffolds into devices is difficult because the available surface area for optoelectronic processes is limited to the scaffold surface. For maximum solar-cell performance, nanowire arrays should present large surface areas for efficient charge transfer to the hole or electron transport layers. Perovskite nanowire films are being explored as active layers for optoelectronic devices, including photodetectors,^16^ lasers,^17^ light-emitting diodes,^18,19^ photovoltaics,^18,20−23^ and field-effect transistors.^24^ Their inherently large active surface areas facilitate fast charge transfer and also improve strain relaxation from thermal expansion during working cycles.^25−27^ Furthermore, each nanowire comprises a single crystal, eliminating grain boundaries that act as charge recombination sites.^28^ Inability to control nanowire orientation, however, has limited their performance as optoelectronic active layers. While each nanowire itself is a single crystal, charges must hop across nanowires in randomly oriented arrays, limiting overall current flow.^29^

We recently developed a strategy to use nanoconfining scaffolds to direct the crystallization of molecular semiconductors into vertical crystal arrays with large exposed surface areas by allowing crystal growth to proceed above the scaffold surface.^30,31^ Here, we build on this approach to grow vertically oriented, unconfined FAPbI_3_ nanowire arrays in which the optoelectronically active α-phase is stabilized. Nanoporous scaffolds comprising vertically aligned cylindrical nanopores were made from anodized TiO_2_, which itself acts as an electron transport layer in MHP solar cells. Using a two-step deposition method, PbI_2_ was first infiltrated in TiO_2_ nanopores and then converted into FAPbI_3_ by immersion in a solution containing FAI. During immersion in FAI solutions containing 25 mol % BAI relative to FAI, stable α-FAPbI_3_ nanowires grew at room temperature, without any need for thermal annealing. When incorporated into solar cells, FAPbI_3_ nanowire arrays achieved short-circuit current densities (*J*_SC_’s) approaching those of top performing FAPbI_3_ bilayer solar cells.

## Results and Discussion

**Scheme 1:**
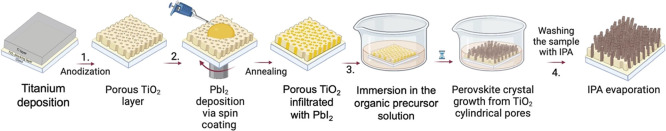
Schematic of the Fabrication Procedure: (1) TiO_2_ Anodization, (2) PbI_2_ Deposition, (3) Immersion in Organic Precursor Solution, and (4) Isopropyl Alcohol (IPA) Evaporation

FAPbI_3_ nanowire arrays were grown from anodized TiO_2_ scaffolds, exhibiting vertically aligned cylindrical nanopores deposited on a transparent fluorinated tin oxide (FTO) electrode. Scheme 1 displays the sample preparation procedure. 100 nm titanium was thermally evaporated onto glass slides (or compact TiO_2_-coated FTO/glass when making solar cells) and then anodized to form transparent porous TiO_2_ scaffolds with vertically aligned cylindrical nanopores (*d*_pore_ = 50 nm; Figure S1a,b). Samples were then annealed for 30 min at 500 °C to convert TiO_2_ to the anatase phase (Figure S1c). Solutions of 0.5 M PbI_2_ in *N,N*-dimethylformamide (DMF) were spin coated onto TiO_2_ scaffolds and annealed at 100 °C for 1 h to evaporate residual DMF. PbI_2_-infiltrated TiO_2_ scaffolds were then immersed in either a 0.5 M solution of formamidinium iodide (FAI) or a 0.5 M solution of FAI:butylammonium iodide (BAI) in a 3:1 molar ratio in isopropyl alcohol (IPA) for times ranging from 30 to 1800 s to convert PbI_2_ to FAPbI_3_. Samples were then rinsed with neat IPA and dried at room temperature.

**Figure 1:**
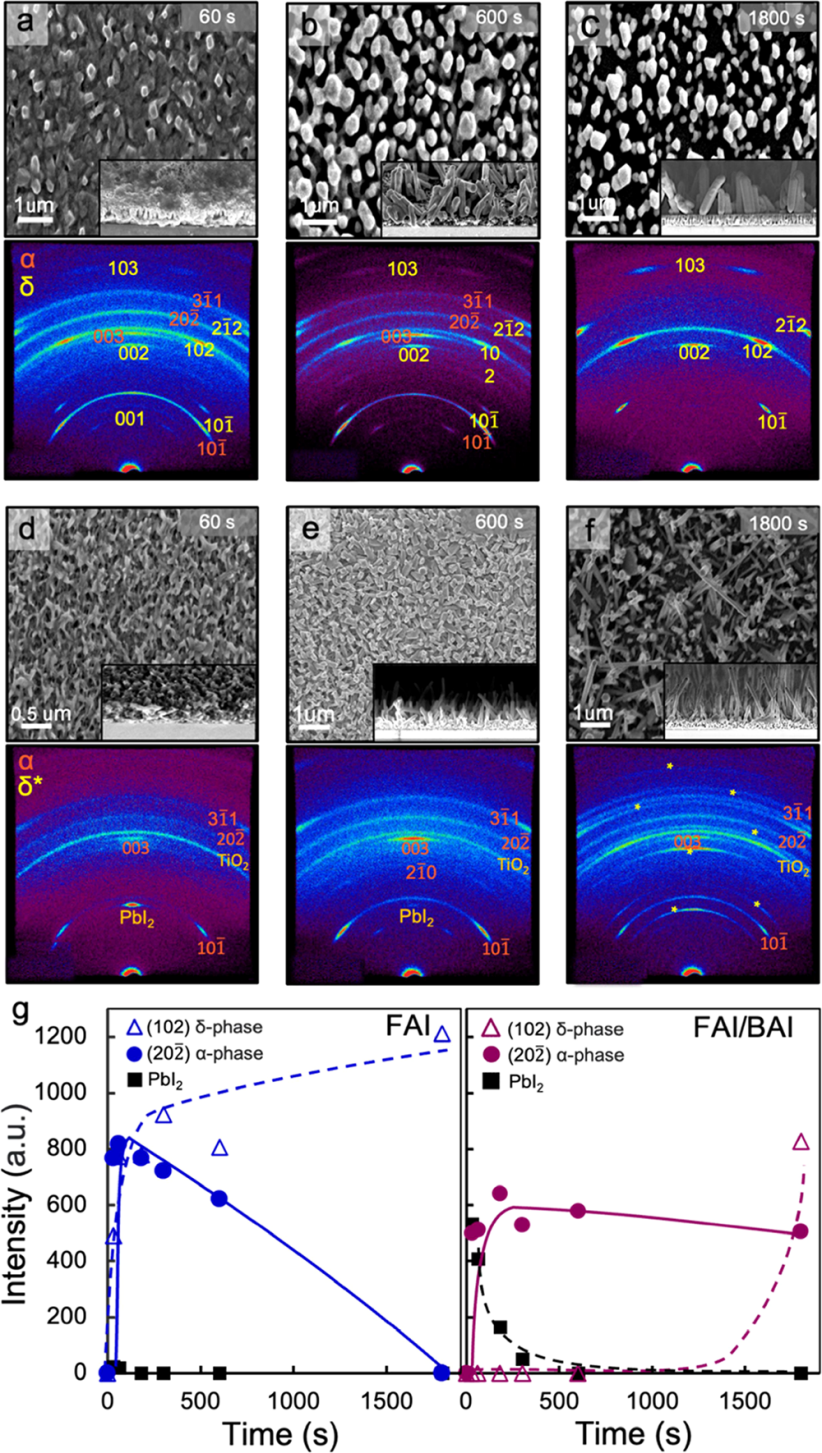
Top-view SEM images and corresponding 2D X-ray diffraction patterns of PbI_2_-infiltrated anodized TiO_2_ scaffolds after immersion in (a–c) 0.5 M formamidinium solution in IPA for 60, 600, and 1800 s, respectively, and (d–f) cosolution of FAI and BAI (3:1 molar ratio) in IPA for 60, 600, and 1800 s, respectively. Cross-sectional SEM images are provided as insets. (g) Time evolution of XRD peak intensities associated with PbI_2_, α-FAPbI_3_, and δ-FAPbI_3_ for the two different immersion conditions.

Upon spin coating, PbI_2_ crystals formed both within TiO_2_ nanopores and on top of the scaffolds, preferentially orienting with the (001) plane parallel to the substrate surface (Figure S2). Figure 1a–c displays SEM images and corresponding 2D X-ray diffraction patterns of PbI_2_-infiltrated TiO_2_ scaffolds after immersion in an FAI solution for 60, 600, and 1800 s. After 60 s of immersion in the precursor solution, the sample surface roughened due to the formation of ∼300 nm grains. These grains grew larger with longer immersion times, reaching diameters of 500 ± 30 and 700 ± 50 nm at 600 and 1800 s, respectively. For immersion times longer than 3600 s, the nanowires began to dissolve. Cross-sectional SEM images revealed these grains to be nanowires growing vertically from the scaffold surface (Figure 1b,c insets). In comparison, immersing a PbI_2_ film spin coated on a flat glass substrate in an FAI solution resulted in micron-scale FAPbI_3_ crystals oriented with their long axes parallel to the substrate surface (Figure S3). These results indicate that the nanoporous TiO_2_ scaffold directs FAPbI_3_ crystallization into vertically oriented nanowire arrays.

We previously observed such scaffold-directed nanowire formation in organic semiconducting triisopropylsilylethynyl pyranthrene^32^ and perylene^33^ crystals. When nucleation occurs within the scaffold, nuclei oriented with their fast growth direction parallel to the unconfined direction of the nanopore achieve the critical nucleus size more quickly than misoriented nuclei, resulting in preferential orientation of nanoconfined crystals.^34^ Preferred orientation of crystals grown in nanoconfined spaces has been observed for other compounds, including glycine,^35^ ferroelectric polymers like P(VDF-TrFE),^36^ and poly(ethylene oxide)s.^37^ In cases where crystallization proceeds beyond the scaffold surface, nanowire arrays with large exposed surface areas can be achieved. We also expect Ostwald ripening to occur during solvent evaporation,^30^ resulting in significantly larger nanowire diameters compared to the average nanopore diameters of 50 nm in the scaffold.

2D XRD patterns were collected on FAI-immersed samples for different immersion times to monitor the conversion process. After 60 s of immersion in FAI solution, the 001 reflection of PbI_2_ almost completely disappeared from the XRD pattern (Figure 1a), while other diffraction spots appeared. These spots were indexed to the α and δ phases of FAPbI_3_ (labeled in red and yellow, respectively). These diffraction peaks display some texturing, with the 00l reflections of both crystal phases exhibiting the strongest intensity along *q_xy_* = 0 Å^–1^. After 600 s of immersion, the 001 reflection of PbI_2_ was absent from the XRD pattern. After 1800 s, only reflections associated with δ-FAPbI_3_, the thermodynamically stable phase at room temperature,^38^ were present. With the exception of the 101 reflection of anatase TiO_2_, which appears as an isotropic ring, other reflections associated with the perovskite phases exhibit strong texturing in the azimuthal direction. Nonuniform intensity along the azimuthal direction at a given q value indicates a highly preferred orientation with the (001) plane parallel to the substrate surface (Figure 1c). 89 ± 12° as the average tilt angles of nanowires, calculated from cross-section SEM images of FAI 1800 s immersed samples (Figure S4a,b), also confirms vertical growth. Upon close examination of the SEM image in Figure 1c, the nanowires adopt a hexagonal cross-section, consistent with the hexagonal *P*6_3_*mc* symmetry of δ-FAPbI_3_, in which the 6-fold axis lies perpendicular to the (001) plane (Figure S5).

The formation of α-FAPbI_3_ during immersion of PbI_2_ in FAI solutions is surprising because δ-FAPbI_3_ is the thermodynamically stable polymorph at room temperature.^39,40^ To achieve the α-phase, a thermal annealing step above 150 °C is typically required.^41^ From a thermodynamic standpoint, the stable phase at a given temperature has the lowest Gibbs free energy, *G(T)*. *G(T)* is a sum of the surface and volume free energies, with surface energy contributions becoming significant for nanoscale crystals with large surface area-to-volume ratios.^42^ Polymorph transition temperatures at which *G(T)* of the two phases are equal can thus shift when comparing bulk crystals to nanocrystals.^43,44^ Here, α-FAPbI_3_ is present at early immersion times when the surface area-to-volume ratio of the crystals is large. As the crystals grow with increasing immersion time, the volume free energy contribution to *G(T)* likewise increases, at some point rendering δ-FAPbI_3_ as the thermodynamically stable phase.

Based on a previous report that the presence of small amounts of BAI in solution can stabilize α-FAPbI_3_ from conversion to δ-FAPbI_3_,^45^ we performed the same experiments using a 3:1 molar ratio of FAI/BAI at a total concentration of 0.5 M in IPA. Figure 1d–f displays SEM images and corresponding 2D XRD patterns of PbI_2_-infiltrated anodized TiO_2_ scaffolds after immersion in the FAI/BAI solution for 60, 600, and 1800 s. Similar to the sample immersed in pure FAI solution, the sample surface was roughened with longer immersion times through nanowire growth. These nanowires exhibited larger aspect ratios compared to those grown in pure FAI solution, reaching heights and diameters of 300 ± 20 and 50 ± 10 nm, respectively, at 600 s of immersion and 1.0 ± 0.2 and 100 ± 20 nm, respectively, at 1800 s of immersion. Nanowire tilt angles of 87 ± 13° were measured after 1800 s of immersion in an FAI/BAI solution (Figure S4c,d).

2D XRD patterns of these samples revealed that reflections associated with PbI_2_ decreased in intensity with increasing immersion time and were completely absent from the XRD pattern collected at 1800 s. In the patterns collected at 60 and 600 s of immersion, other diffraction peaks were indexed to α-FAPbI_3_ only. After 1800 s of immersion, peaks associated with δ-FAPbI_3_ were also present.

Figure 1g displays a comparison of the time evolution of the 001, 202̅, and 102 reflection intensities of PbI_2_, α-FAPbI_3_, and δ-FAPbI_3_, respectively, for samples immersed in FAI and FAI/BAI solutions. PbI_2_ conversion occurred much more rapidly when immersed in FAI solution compared to the FAI/BAI solution. Conversely, δ-FAPbI_3_ formation was significantly delayed when immersion occurred in the presence of BAI. This delay may be attributed to BA^+^ interactions with FAPbI_3_ crystals that affect both the thermodynamics and the kinetics of crystal growth. Long-chain alkyl and aromatic ammonium cations have previously been reported to bond to α-FAPbI_3_ crystal surfaces, reducing the surface energy and lowering *G(T)*.^45^ Ex situ SEM images displayed in Figure 1a–f also indicate that crystal growth is slower in FAI/BAI solutions compared to FAI solutions, resulting in a longer time period of immersion, up to 600 s, during which α-FAPbI_3_ is stable and the only phase present. Vertical nanowire growth is thus attributed to the presence of a nanoporous scaffold, while α-phase stabilization is primarily attributed to the presence of BAI.

**Figure 2:**
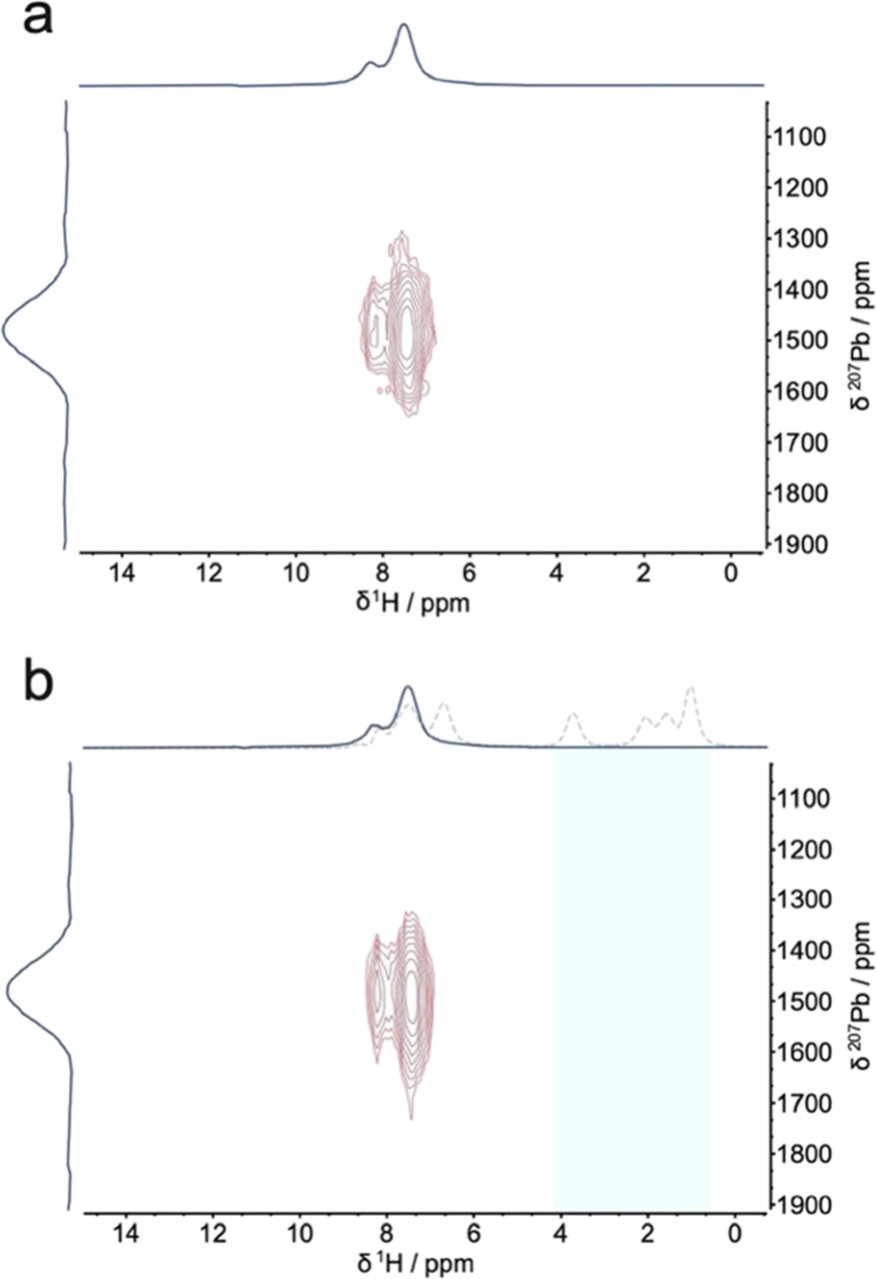
Solid-state NMR. ^1^H detected ^207^Pb → ^1^H HETCOR for (a) FAPbI_3_ and mechanosynthesized (b) BA_2_FA_*n*–1_Pb_*n*_I_3*n*+1_ (*n* = 7) (solid line) with overlaid ^1^H echo spectrum (dashed line). Shaded area calls attention to the absence of ^207^Pb correlation with BA associated ^1^H nuclei. Experiments were conducted under 40 kHz magic angle spinning and with a cross-polarization (CP) contact time of 4 ms.

Peaks associated with 2D perovskite BA_2_PbI_4_ or quasi-2D perovskites were not observed in the XRD patterns of samples immersed in FAI/BAI cosolutions, suggesting that BA cations do not incorporate into the crystal lattice of nanowires. To independently assess the ability of BAI to incorporate into FAPbI_3_ crystals at a molar ratio of 3:1 FAI:BAI, we performed solid-state nuclear magnetic resonance (ss-NMR) spectroscopy. Whereas XRD relies on the regular, periodic arrangement of molecules in 3D space to reveal crystal structures, ss-NMR probes local molecular environments and can thus detect BAI incorporation. Prohibitively small sample volumes unfortunately precluded direct ss-NMR measurements on FAPbI_3_ nanowires grown on TiO_2_ scaffolds in the absence and presence of BAI. Instead, we collected ss-NMR spectra on mechanosynthesized MHPs using the same stoichiometric ratios used in nanowire growth. Previous studies have found mechanosynthesized MHPs to adopt the same structure as solution-processed MHPs.^46^Figure 2 displays ^1^H detected ^207^Pb → ^1^H HETCOR ss-NMR spectra of mechanosynthesized 3D perovskite FAPbI_3_ and BA_2_FA_*n*–1_Pb_*n*_I_3*n*+1_ (*n* = 7). The ^207^Pb → ^1^H HETCOR experiment relies on magnetization transfer between the ^207^Pb and ^1^H nuclei in order to observe a correlation in the 2D spectrum. The spectrum in Figure 2 shows correlation peaks between the ^1^H signals of formamidinium at 8.3 ppm (NH_2_) and 7.5 ppm (CH) and the perovskite ^207^Pb signal at 1477 ppm. No correlation is observed for ^1^H resonances pertaining to ^1^H nuclei in butylammonium.^47^ This result indicates a lack of BA incorporation into the perovskite lattice for *n* = 7. In addition, the ^207^Pb resonance is indistinguishable for *n* = 7 and the 3D perovskite, indicating a similar magnetic environment for ^207^Pb nuclei. Though 2D hybrid perovskites based on methylammonium, such as BA_2_MA_*n*–1_Pb_*n*_I_3*n*+1_, have been shown to incorporate butylammonium into the lattice for at least up to *n* = 2,^47^ hybrid perovskites based on formamidinium do not form readily above *n* = 2.^48,49^

**Figure 3:**
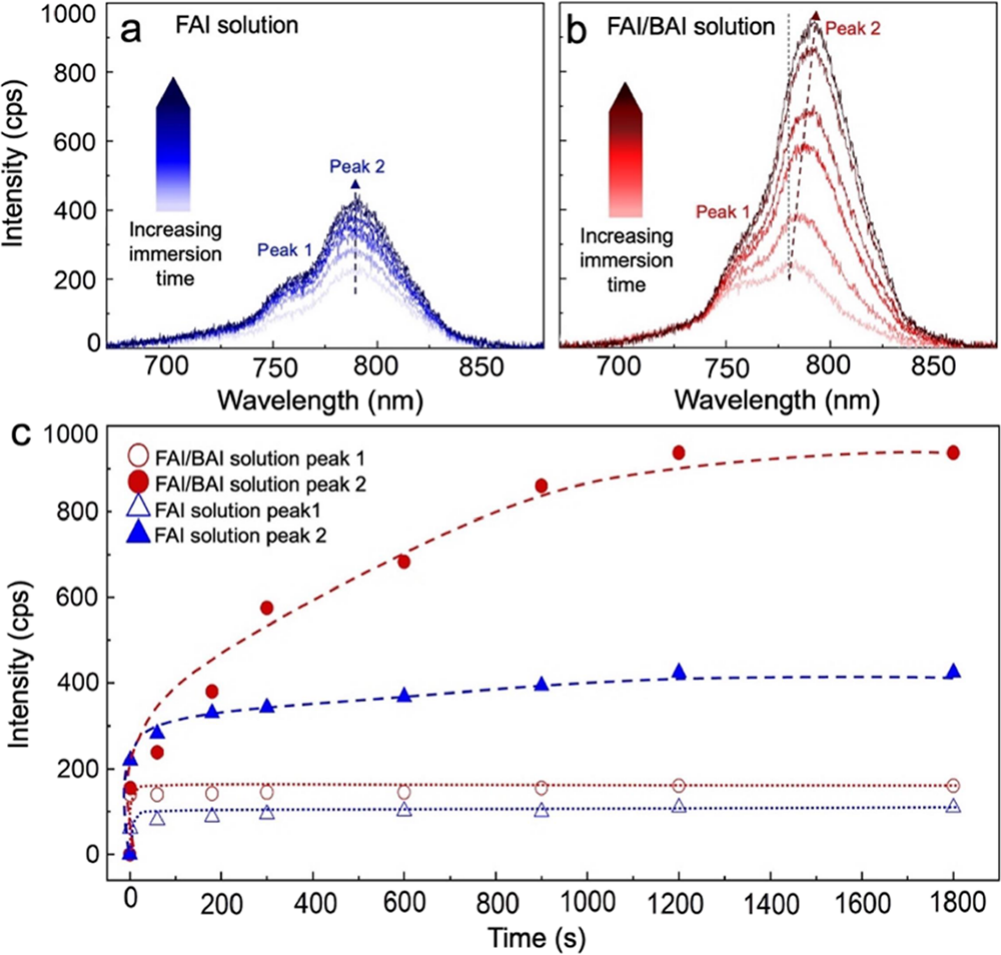
Time-dependent in situ PL spectra collected on PbI_2_-infiltrated TiO_2_ scaffolds at 1, 60, 90, 300, 600, 900, and 1200 s of immersion in (a) FAI and (b) FAI/BAI solutions, respectively (λ_ex_ = 532 nm). (c) Peak intensity evolution over time for samples immersed in FAI and FAI/BAI solutions.

Crystal growth kinetics was monitored in situ by collecting photoluminescence (PL) spectra during solution immersion. PbI_2_ and α-FAPbI_3_ exhibit PL peaks around 500 nm^50−52^ and 800 nm,^53,54^ respectively, and are thus easily distinguishable from one another. Figure 3a,b displays the PL spectra collected on PbI_2_-infiltrated TiO_2_ scaffolds after 1, 60, 90, 300, 600, 900, and 1200 s of immersion in FAI and FAI/BAI solutions, respectively (λ_ex_ = 532 nm). After just 1 s of immersion, two peaks centered around 760 and 790 nm appeared in the PL spectra of both samples. The lower energy peak (Peak 2) is associated with α-FAPbI_3_.^55−57^ For both samples, the intensity of Peak 1 did not change significantly with longer immersion times. The intensity of Peak 2, on the other hand, increased significantly for the sample immersed in an FAI/BAI cosolution.

Gaussian curves were fit to each spectrum to decouple the contributions of the high (Peak 1) and low (Peak 2) energy peaks to the overall spectra (Figures S6 and S7). Peak intensities versus time extracted from the Gaussian fits are displayed in Figure 3c. For both sets of samples, Peak 1 reached its maximum intensity by 180 s of immersion with no change at longer immersion times. For the FAI-immersed sample, Peak 2 reached 90% of its maximum value within 300 s of immersion. In contrast, an immersion time of 600 s was needed to reach the same threshold for the FAI/BAI sample. The maximum intensity of Peak 2 was ∼2.2 times larger for the FAI/BAI-immersed sample compared with that of the FAI-immersed sample. Collectively, these results indicate that (1) the presence of BA^+^ decreases the α-FAPbI_3_ crystallization rate and (2) the overall crystallinity achieved is higher compared to the FAI-immersed sample. We hypothesize that BA^+^ cations slow α-FAPbI_3_ growth during early immersion times (<100 s). BA^+^ cations also suppress δ-FAPbI_3_ nucleation, allowing α-FAPbI_3_ crystals to continue growing at longer immersion times.

Comparing ex situ XRD data to in situ PL data, we can infer a more detailed timeline of the crystallization process. Both XRD and PL data confirm that (1) α-FAPbI_3_ forms during immersion in FAI and FAI/BAI solutions, (2) the presence of BAI slows α-FAPbI_3_ crystallization, and (3) the total amount of α-FAPbI_3_ crystals after 20 min is larger for samples immersed in FAI/BAI solution compared to FAI solution. A significant difference between the XRD and PL data sets, however, exists for samples immersed in an FAI solution for longer times. Whereas in situ PL data indicate that the amount of α-FAPbI_3_ crystals does not change appreciably between 300 and 1800 s of immersion, ex situ XRD measurements show that α-FAPbI_3_ completely disappears by 20 min of immersion in FAI solution. These results suggest that α- to δ-phase conversion occurs during film drying.

Previous reports have demonstrated that MHP crystal size can affect the bandgap.^58−60^ Steady-state PL spectra collected on methylammonium lead iodide perovskite (CH_3_NH_3_PbI_3_) samples at different stages of PbI_2_ conversion in a sequential perovskite deposition, for example, revealed a blue shift of the PL spectra and an unusual asymmetry in the emission weighted toward higher energies at the start of the reaction.^53^ This hypsochromic shift is associated with the presence of smaller nanocrystallites in the early stages of perovskite formation. Smaller crystals exhibit larger band gaps as a result of larger distortion of the Pb–I bond.^61,62^

In the present experiments, FAPbI_3_ crystal nucleation and growth starts within the 50 nm cylindrical pores of anodized TiO_2_, followed by subsequent growth above the pores. For crystals grown within the TiO_2_ nanopores, their size is restricted to the pore diameter, resulting in two populations of crystal sizes associated with those crystallized within and above the scaffold, respectively. Based on the time evolution of the peaks, we speculate that Peak 1 is from PL emission of nanoconfined α-FAPbI_3_ crystals that form at early immersion times while Peak 2 represents emission from larger crystals formed above the TiO_2_ scaffold surface at longer immersion times. Peak 2 of the FA/BA immersed sample (Figure 3b) also exhibited a small red shift with increasing immersion time from 781 to 793 nm, indicated by the dashed red arrow. This bathochromic shift is consistent with an increasing α-FAPbI_3_ crystal size, also observed in ex situ SEM images (Figure 1 d–f). This red shift is not observed in the PL spectra of the sample immersed in FAI solution, consistent with the formation of α-FAPbI_3_ at early immersion times, followed by δ-FAPbI_3_ formation.

**Figure 4:**
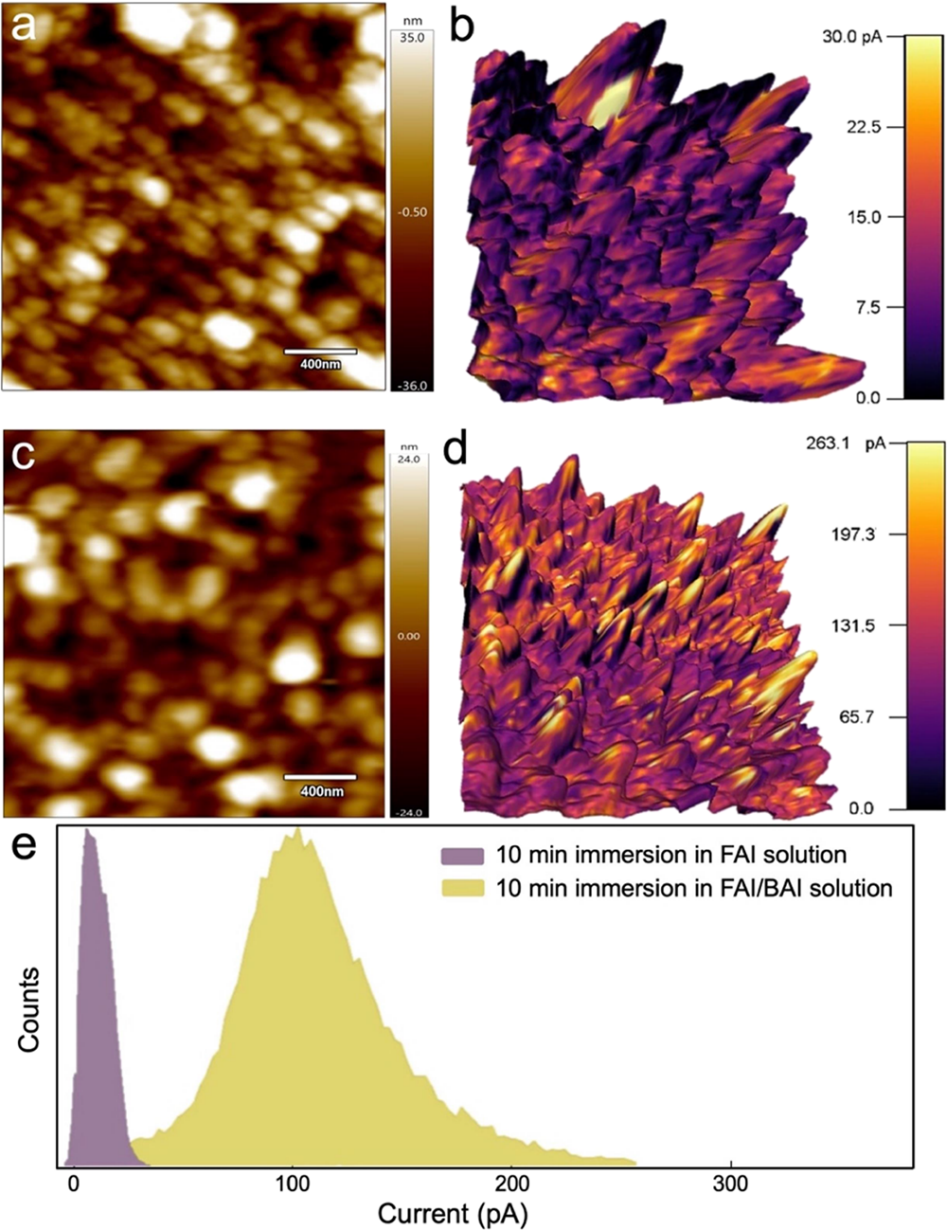
AFM (a, c) height maps and (b, d) conductivity maps overlaid on 3D height maps of FAPbI_3_ nanowires grown for 600 s in FAI and FAI/BAI solutions, respectively. (e) Histograms tabulating the normalized frequencies of 256 × 256 current measurements in conductive AFM maps for each of the four configurations.

Samples immersed in FAI/BAI solution for 600 s exhibiting relatively large α-FAPbI_3_ nanowires without any δ-FAPbI_3_ present were selected for further optoelectronic characterization and compared with samples immersed in FAI solution for the same amount of time. Figure 4 displays atomic force microscopy (AFM) height and conductivity maps of PbI_2_-infiltrated TiO_2_ samples after immersion in FAI (Figure 4a,b) and FAI/BAI (Figure 4c,d) solutions for 600 s and subsequent drying in air. For these experiments, TiO_2_ scaffolds were fabricated on conductive fluorine-doped tin oxide (FTO)-coated glass, which served as the bottom electrode (see Experimental Section).

In both samples, vertically oriented nanowires were observed in the height maps. Conductivity maps overlaid on these height maps revealed the tips of nanowires in the FAI-immersed sample to be insulating. Nanowire tips likely comprise δ-FAPbI_3_, the insulating nonperovskite phase, which forms at longer immersion times in FAI solution. Nanowires formed in an FAI/BAI solution, on the other hand, exhibit maximum conductivity at the nanowire tips, indicating that these nanowires comprise only α-FAPbI_3_. Corresponding histograms of the current values for these two samples (256^2^ measurements for each sample) are displayed in Figure 4e. The average conductivity for the FAI-immersed sample was 8 pA compared to 104 pA for the FAI/BAI-immersed sample. The significantly higher conductivity of the FAI/BAI-immersed sample is due to the suppression of δ-FAPbI_3_ during immersion.

**Figure 5:**
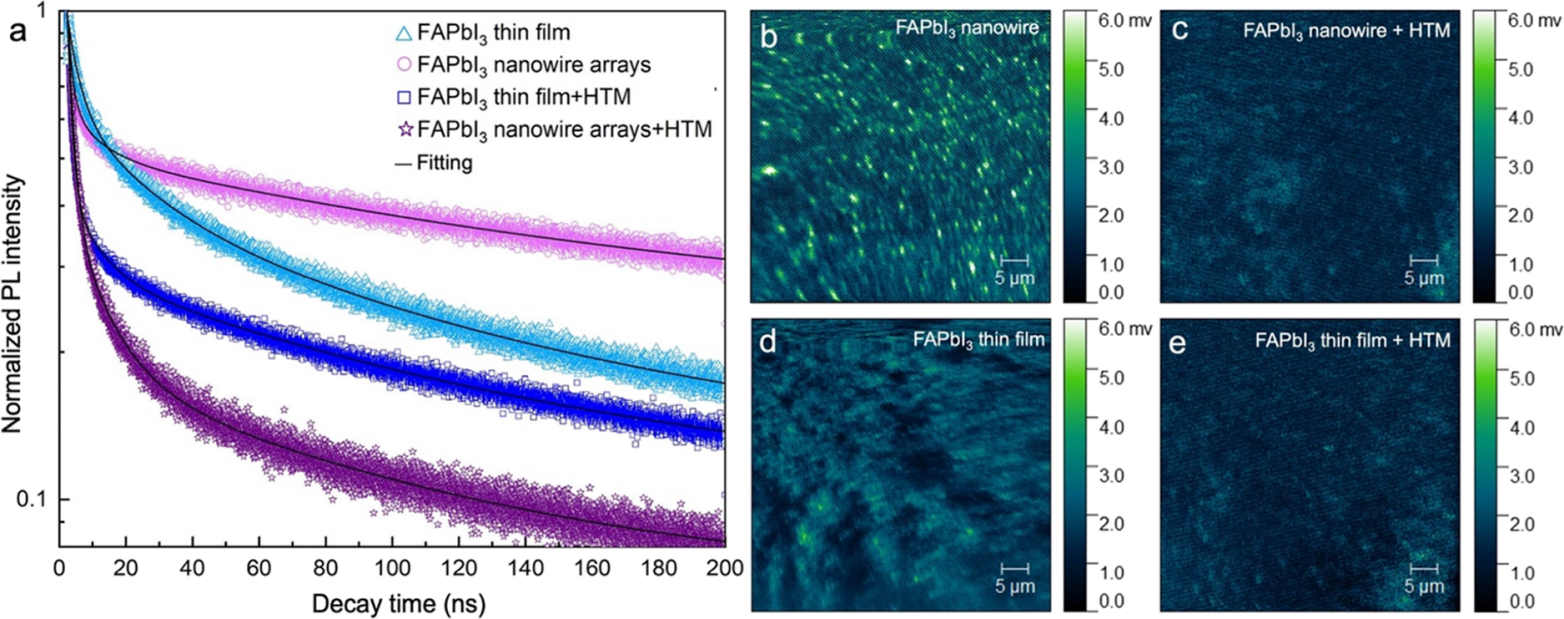
(a) Normalized time-resolved photoluminescence (TRPL) curves of a FAPbI_3_ nanowire array and a thin film before and after Spiro-MeOTAD deposition. (b, c) Hyperspectral PL map of perovskite nanowire arrays before and after Spiro-MeOTAD deposition, respectively. (d, e) Hyperspectral PL maps of perovskite thin films before and after Spiro-MeOTAD deposition, respectively.

Time-resolved photoluminescence (TRPL) measurements were carried out on an FAI/BAI, 600 s immersed sample with and without the presence of Spiro-OMeTAD, a common hole transport material (HTM) used in MHP solar cells. For comparison, TRPL measurements were also collected on a FAPbI_3_ thin-film spun cast onto a porous anodized TiO_2_ scaffold. Figure 5a displays the PL decay kinetics of FAPbI_3_ nanowire arrays and FAPbI_3_ thin films with and without Spiro-OMeTAD. TRPL curves were fitted to a biexponential decay.
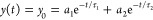
1

Time constants τ_1_ and τ_2_ represent fast and slow recombination pathways, respectively, and *a*_1_ and *a*_2_ represent their relative contributions to the overall decay kinetics. FAPbI_3_ nanowires without Spiro-OMeTAD exhibited τ_1,nw_ and τ_2,nw_ values of 2.7 and 99.9 ns, respectively. Fast recombination dominated the decay kinetics with *a*_1,nw_ = 1.3 (78% contribution) and *a*_2,nw_ = 0.4 (22% contribution). For FAPbI_3_ thin films, τ_1,film_ = 5.2, τ_2,film_ = 63.2 ns, *a*_1,film_ = 0.79 (63% contribution), and *a*_2,film_ = 0.5 (37% contribution). Fitted parameters are summarized in Table S1.

Slow PL decay is typically associated with radiative recombination in the bulk, while fast decay is attributed to surface defect-mediated nonradiative recombination.^63−66^ Slower bulk recombination in FAPbI_3_ nanowires compared to the spun cast film (i.e., τ_2,nw_ > τ_2,film_) indicates that the nanowires exhibit a higher degree of crystallinity and fewer defects within the crystals, likely due to slower crystallization of nanowires compared to crystals formed during spin coating. On the other hand, fast decay contributes to a larger percentage of the overall decay kinetics in the nanowires compared to the thin film (78 versus 63%, respectively) due to the significantly larger surface area of nanowires compared to thin films.

For both samples, the presence of Spiro-OMeTAD significantly decreased the PL lifetimes due to hole transfer from FAPbI_3_ to Spiro-OMeTAD.^18,67^ PL decay kinetics were dominated by fast decay with *a*_1,nw+HTM_ = 3.1 (92.8% contribution) and *a*_1,film+HTM_ = 3.3 (93.5% recombination), indicating efficient charge transfer between FAPbI_3_ and Spiro-OMeTAD in both samples. τ_2,film+HTM_ remained unchanged at 63.3 ns compared to that of τ_2,film_, while τ_2,nw+HTM_ decreased significantly to 35 ns. The large decrease in slow PL decay attributed to excited states generated in the nanowire interior suggests efficient charge diffusion to nanowire/Spiro-OMeTAD interfaces due to high surface area-to-volume ratios.

Figure 5b–e displays hyperspectral PL maps of a FAPbI_3_ nanowire array and a spun cast thin film before and after Spiro-OMeTAD deposition collected at λ_em_ = 800 nm using an excitation wavelength, λ_ex_ = 532 nm. The laser line was filtered using 650 nm long-pass filter. Strong PL signals were observed from the nanowire tips (Figure 5b). The PL map of the thin film exhibited larger domain sizes and less intensity in comparison (Figure 5d). The presence of Spiro-OMeTAD quenched the PL signal in both samples (Figure 5c,e), consistent with the TRPL data. Interestingly, the hyperspectral PL map of a FAPbI_3_ nanowire array grown during immersion in an FAI-only solution for 600 s displayed dark spots corresponding to the nanowire tips (Figure S9). These results are consistent with the conductive AFM map in Figure 4b, which suggested the presence of insulating δ-FAPbI_3_ at the nanowire tips in this sample.

**Figure 6:**
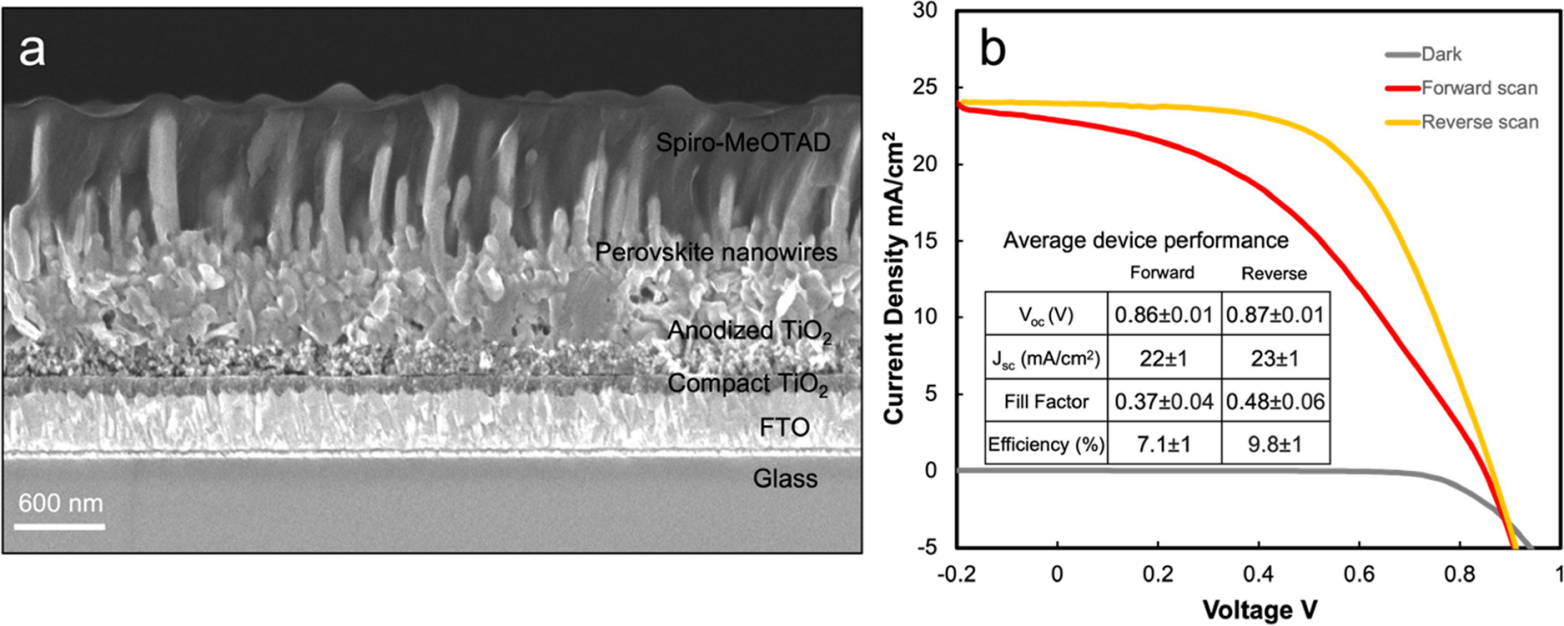
(a) Cross-sectional SEM image of a FAPbI_3_ nanowire solar cell before top gold electrode deposition. (b) *J*–*V* curve of the best-performing FAPbI_3_ nanowire solar cell under AM 1.5 illumination at an intensity of 100 mW/cm^2^. Table summarizes average values and standard deviations for solar-cell parameters measured for five devices.

FAPbI_3_ nanowire solar cells were fabricated using FTO as the bottom electrode, anodized TiO_2_ as the electron transport layer, FAPbI_3_ nanowires formed through PbI_2_ immersion in FAI/BAI solution for 600s as the light absorbing layer, Spiro-OMeTAD as the hole transporting layer, and gold as the top electrode. Figure 6a displays a cross-sectional SEM image of a FAPbI_3_ nanowire solar cell with each layer labeled. Spiro-OMeTAD infiltrated the nanowire array completely, forming a continuous layer spanning from anodized TiO_2_ to the FAPbI_3_ nanowire tips. Current density–voltage (*J*–*V*) characteristics for devices with active areas of 4.2 mm^2^ were measured under AM 1.5 illumination. Figure 6b displays the *J*–*V* curve of the best performing FAPbI_3_ nanowire solar cell. The inset table provides solar-cell parameters averaged over five devices. An open-circuit voltage (*V*_OC_) of 0.87 V, short-circuit current density (*J*_SC_) of 23.9 mA/cm^2^, fill factor (FF) of 0.56, and photoconversion efficiency (PCE, η) of 11.7% were measured. While these PCE values are lower than record efficiencies of >21% for FAPbI_3_ solar cells,^3,4^ we expect further improvements can be achieved through optimization of the electron and hole transport layers. Promisingly, the *J*_SC_ values of FAPbI_3_ nanowire solar cells are approaching the highest reported values for FAPbI_3_ solar cells of 26–27 mA cm^–2^.^4,68−73^ Enhanced *J*_SC_ values for nanowire-based photovoltaic devices compared to their thin-film counterparts have been reported for silicon solar cells due to larger available surface areas for charge transfer.^74−76^ Furthermore, α-FAPbI_3_ formed directly during PbI_2_ immersion in FAI and FAI/BAI solutions, precluding the need for a high temperature annealing step to convert δ-FAPbI_3_ to α-FAPbI_3_.

## Conclusions

Compared to spun cast thin films, vertically oriented nanowire arrays provide large surface areas for fast charge transfer and continuous single-crystal domains for charge transport to electrodes. Here, anodized TiO_2_ films with cylindrical nanopores were used to direct the conversion of PbI_2_ into FAPbI_3_ during immersion in an FAI solution. With crystal growth initially constrained in two dimensions in the TiO_2_ nanopores, the FAPbI_3_ nanowire growth proceeded vertically from the underlying substrate surface. In the case of FAPbI_3_, an additional advantage of nanowire growth via solution immersion in the presence of BAI is α-phase stabilization at room temperature, which will drive down manufacturing costs by precluding the need for energy-intensive thermal annealing of the active layer. Because the TiO_2_ scaffolds used to direct FAPbI_3_ crystallization are themselves commonly used as electron transport layers in organic and perovskite solar cells, scaffold removal was not necessary to fabricate the working devices. Scaffold-directed crystallization of organic and hybrid compounds thus presents a promising strategy to achieve optimized crystal orientations and sizes during rapid solution processing.

## Experimental Section

### TiO_2_ Scaffold Fabrication

100 nm of titanium metal was deposited on a glass substrate or FTO-coated glass slide by thermal evaporation. The Ti layer was anodized in a 0.38 vol % NH_4_F in ethylene glycol solution at 25 °C for 3 min at 50 V, followed by thermal annealing at 450 °C for 30 min.

### FAPbI_3_ Nanowire Growth

40 μL of 0.5 M PbI_2_ in DMF was spin coated onto TiO_2_ scaffolds at 2000 rpm for 30 s followed by annealing at 100 °C for 1 h. PbI_2_-infiltrated TiO_2_ samples were then immersed in 0.5 M FAI or FAI/BAI (3:1 molar ratio) solutions in IPA for times ranging from 15 to 3600 s. Samples were removed from the solutions, rinsed with neat IPA for 5 s, and left at room temperature to dry. All steps were performed in a N_2_-filled glovebox.

### FAPbI_3_ Mechanosynthesis

3D FAPbI_3_ perovskites were synthesized via mechanosynthesis using a Retsch MM 400 electric mixer mill. The starting materials, PbI_2_ (Sigma-Aldrich) and FAI (Sigma-Aldrich), were weighed accordingly to yield 1 g of product and then added to a 10 mL agate jar containing an agate ball (10 mm diameter) and ground for 60 min at a 25 Hz mixing rate. The resulting powder was then annealed in air a tube furnace at 150 °C for 60 min. The 2D perovskites were synthesized following the same method, with BAI (Great Cell Solar Materials) being added to the reaction mixture. The FAI:BAI:PbI_2_ molar ratios used to achieve *n* = 7 were 6:2:7.

### FAPbI_3_ Thin Film Deposition

A 1 M cosolution of formamidinium iodide (FAI) and PbI_2_ in *N,N*-dimethylformamide (DMF) was prepared and 30 μL of it spun cast onto an anodized TiO_2_ scaffold at 2000 rpm for 30 s. The sample was annealed at 120 °C for 30 min.

### Structural Characterization

Samples were imaged using a high-resolution field emission scanning electron microscope operated at 3 kV and 100 pA (Carl Zeiss HR-SEM). X-ray diffraction patterns were collected using a Bruker AXS D8 Discover GADDS microdiffractometer with a Cu–Kα source. The X-ray beam spot was elliptical, with an approximate average diameter of 800 μm.

### Solid-State NMR Characterization

^1^H and ^207^Pb NMR spectra were recorded on a 750 MHz Wide-Bore Bruker spectrometer equipped with an Avance Neo Console and a 1.9 mm three-channel variable-temperature magic angle spinning probe. Mechanosynthesized samples were packed into 1.9 mm zirconia rotors and spun at up to 40 kHz with active cooling using a Bruker BCU II unit set to 240 K. ^1^H detected (^207^Pb → ^1^H) 2D heteronuclear correlation (HETCOR) spectra were collected using a delay time of 0.5 s, a π/2 pulse length of 3.45 μs, and a mixing time of 4 ms.

### In Situ Microphotoluminescence (μPL) Spectroscopy

μPL measurements were collected in air at room temperature using a home-built confocal microscope setup. Samples were excited by a continuous wave 532 nm laser diode. A laser spot size of ∼1 μm was achieved using a microscope objective with a numerical aperture of 0.42. The relative position between the sample and the laser spot was adjusted with a piezoelectric *xyz* actuator. Laser stray light was removed with a 550 nm long-pass filter. The μPL emission from the sample was collected in a multimode fiber and dispersed using a 0.75 m focal length spectrometer equipped with a liquid nitrogen cooled silicon CCD camera during the immersion step. A photograph of the PL setup is displayed in Figure S8.

### TRPL Analysis and Hyperspectral PL Mapping

Samples were mounted on a piezo stack, which was used for scanning the sample under an objective. Photoluminescence signal from the sample was collected using an objective (NA = 0.82) coupled to a multimode optical fiber to send the signal to an avalanche photodiode (APD). PL maps were collected with a 532 nm laser and a power of 10 μW. A 550-pass filter was used to filter the laser line. For TRPL measurements, a 635 nm laser operating at 2.5 MHz repetition rate and the power of ∼2 μW was used to excite the sample, and time-correlated single photon counting (TCSPC) was carried out with a coincidence counter (PicoQuant) and an APD. TRPL and PL mapping analyses were performed in air.

### Conductive AFM

Height and conductive AFM (c-AFM) images were collected using an Asylum Research Jupiter XR AFM. The AFM was operated in contact mode using Ti/Ir (5/20 nm)**-**coated AFM tips (model ASYELEC-01-R2) with spring constants between 1.4 and 5.8 N m^–1^. A 1 V bias was applied across the probe and an underlying counter electrode, while the tip was scanned across the nanowire array. Current flow from the electrode to the tip was simultaneously measured, along with height profiles of the nanowires. C-AFM maps were collected in air at room temperature.

### Solar-Cell Fabrication

FTO glass substrates were cleaned by sequential sonication in IPA and acetone for 10 min each. The samples were then exposed to ultraviolet (UV)-ozone for 20 min. A compact TiO_2_ blocking layer was spun cast onto a FTO glass at 4000 rpm for 10 s from a cosolution of 7.75 vol % titanium diisopropoxide and 0.39 vol % 2 M hydrochloric acid in anhydrous ethanol, followed by annealing at 450 °C for 30 min. Anodized TiO_2_ scaffolds were fabricated on the compact TiO_2_ layer following the same method as detailed above. Samples were then treated with UV-ozone for 10 min. FAPbI_3_ nanowires were grown from anodized TiO_2_ scaffolds following the procedure detailed above. A 70 mM solution of 2,2′,7,7′-tetrakis[*N*,*N*-di(4-methoxyphenyl)amino]-9,9′-spirobifluorene (Spiro-MeOTAD) in chlorobenzene with 0.0322 mM solution of tris(2-(1*H*-pyrazol-1-yl)-4-*tert*-butylpyridine)cobalt(III) tri[bis(trifluoromethane) sulfonimide] (FK209), 9.06 mM solution of bis(trifluoromethane)sulfonimide lithium salt (LiTFSI), and 24.42 mM solution of 4-tert-butylpyridine (tBP), was used as the hole transport material. 50 μL of this solution was rapidly dropped in the middle of the substrate while spinning at 4000 rpm for 10 s. 80 nm of gold was thermally evaporated onto the Spiro-MeOTAD layer. All steps were performed in a N_2_-filled glovebox.

### Solar-Cell Characterization

Current density–voltage (*J*–*V*) curves were collected with a Keithley 2636B dual-channel source meter in air at room temperature under AM 1.5 illumination at a light intensity of 100 mW/cm^2^ using a xenon arc lamp (ABET Technologies Model 11002). *J*–*V* curves were obtained with a double voltage sweep between 1 and −0.2 V with a step size of 0.012 V.

## Supporting Information Available

Top-view SEM image of titanium metal deposited on the glass substrate by thermal evaporation; cross-sectional SEM of an anodized TiO_2_ scaffold after PbI_2_ infiltration; top-view SEM image of PbI_2_ film spin coated on a flat glass substrate immersed in FAI solution; SEM cross-section images of two different locations of (a, b) a sample immersed in FAI solution for 1800 s and (c, d) a sample immersed in FAI/BAI solution for 1800 s; crystal structure and simulated single-crystal morphology (generated with Mercury software) of (a) α-phase FAPbI_3_ and (b) δ-phase FAPbI_3_; Gaussian curve fits of in situ PL spectra collected on PbI_2_-infiltrated TiO_2_ scaffolds at 1, 60, 90, 300, 600, 900, and 1200 s of immersion in FAI solution; and Gaussian curve fits for on in situ PL spectra collected on PbI_2_-infiltrated TiO_2_ scaffolds at 1, 60, 90, 300, 600, 900, and 1200 s of immersion in FAI/BAI solution; in situ PL measurement setup; hyperspectral PL map of a FAPbI3 nanowire array; extracted parameters of double-exponential model fitted on TRPL spectra.(PDF)
