# Supplementary material for: Scaffold-Guided Crystallization of Oriented α-FAPbI3 Nanowire Arrays for Solar Cells
Source: ACS Appl Mater Interfaces. 2023 Nov 21;15(48):56127–37. doi: 10.1021/acsami.3c09434 (PMC10711707; doi:10.1021/acsami.3c09434)
Supplement: Supplementary file 1 — am3c09434_si_001.pdf [file am3c09434_si_001.pdf]

Supporting Information

Scaffold-Guided Crystallization of Oriented α-$FAPbI_3$ Nanowire Arrays for Solar Cells

Aida Alaei,[†] *Seyed Sepehr Mohajerani,*[‡] *Ben Schmelmer,*[†] *Thiago I. Rubio,*[†] *Justin Bendesky,*[†] *Min-Woo Kim,*[†] *Sehee Jeong,*[†] *Qintian Zhou,*[†] *Mia Klopfenstein,*[†] *Claudia E. Avalos,*[†] *Stefan Strauf,*[‡] *Stephanie S. Lee,**[†]

[†]*Department of Chemistry and Molecular Design Institute, New York University, New York, NY 10003, USA*

[‡]*Department of Physics, Stevens Institute of Technology, Hoboken, NJ 07030, USA*

*stephlee@nyu.edu

Supplementary Figures

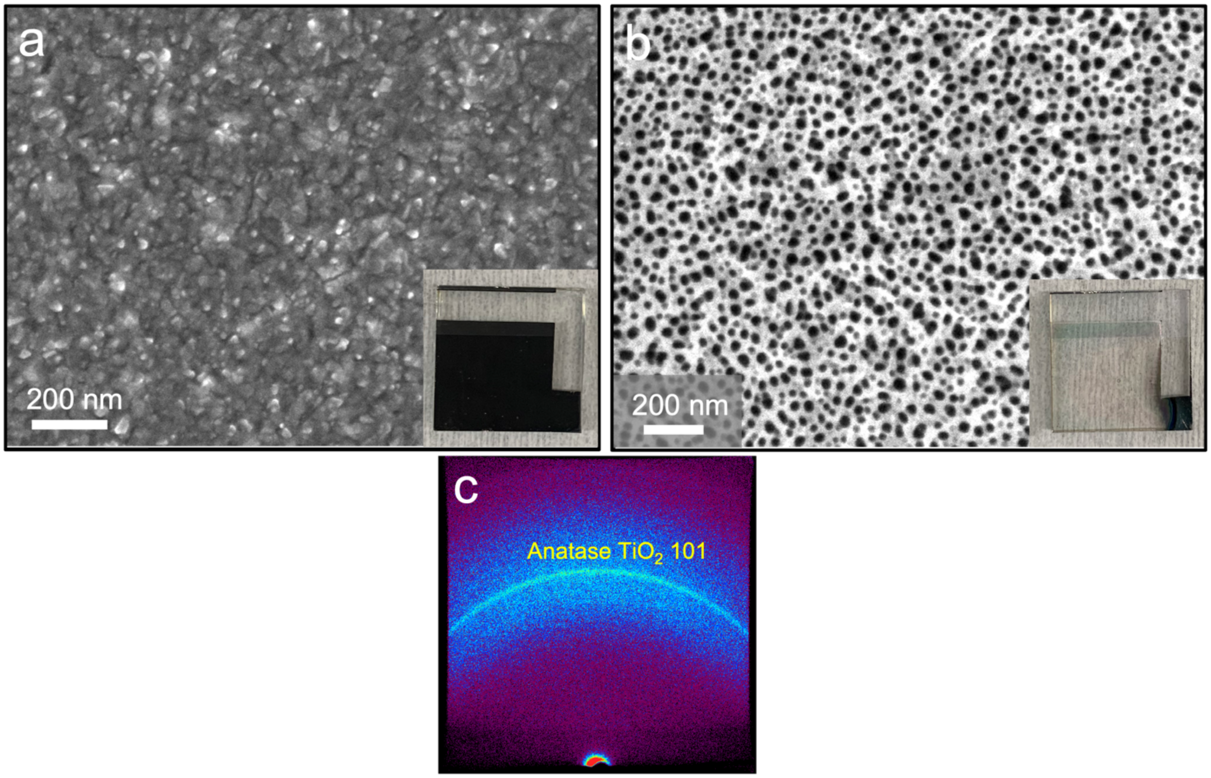

Figure S1. a) Top-view SEM image of titanium metal deposited on glass substrate by thermal evaporation. Inset: Photograph of deposited titanium layer before anodization. b) Top-view SEM image of porous TiO_2 after anodization and annealing. Inset: Photograph of transparent anodized TiO_2 layer. c) corresponding 2D X-ray diffraction patterns of anodized TiO_2 scaffolds after annealing step showing the presence of anatase 101 reflection.

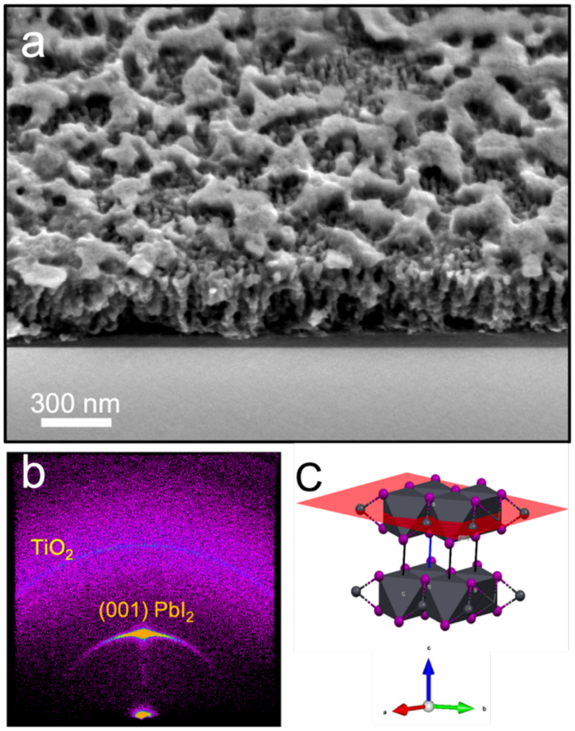

Figure S2. a) Cross sectional SEM of an anodized TiO_2 scaffold after PbI_2 infiltration. b) 2D X-ray diffraction pattern of a PbI_2-infiltrated anodized TiO_2 scaffold. C) Crystal structure of PbI_2 with the (001) plane highlighted in red.

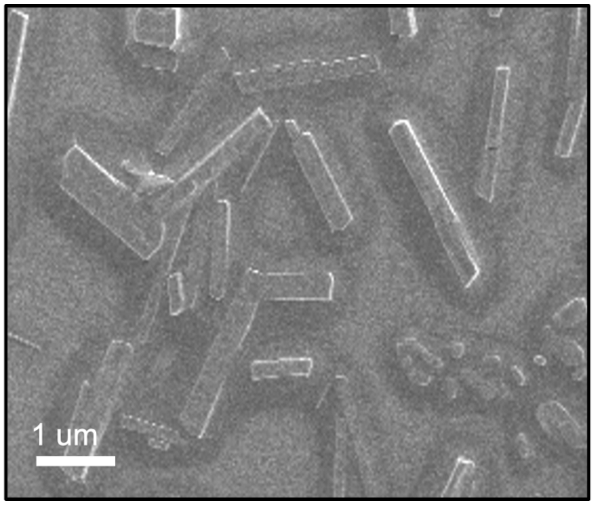

Figure S3. Top-view SEM image of PbI_2 film spin coated on a flat glass substrate immersed in FAI solution.

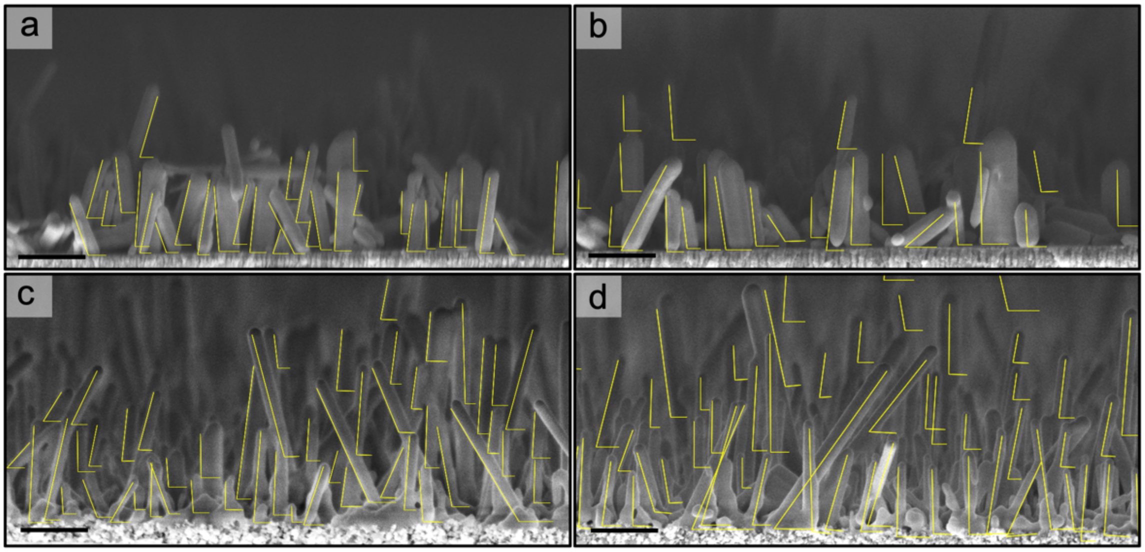

Figure S4. SEM cross section images of two different locations of a and b) a sample immersed in FAI solution for 1800 s and c and d) a sample immersed in FAI/BAI solution for 1800 s. ImageJ was used to calculate the average nanowire tilt angle (yellow lines). Scale bar = 1μm.

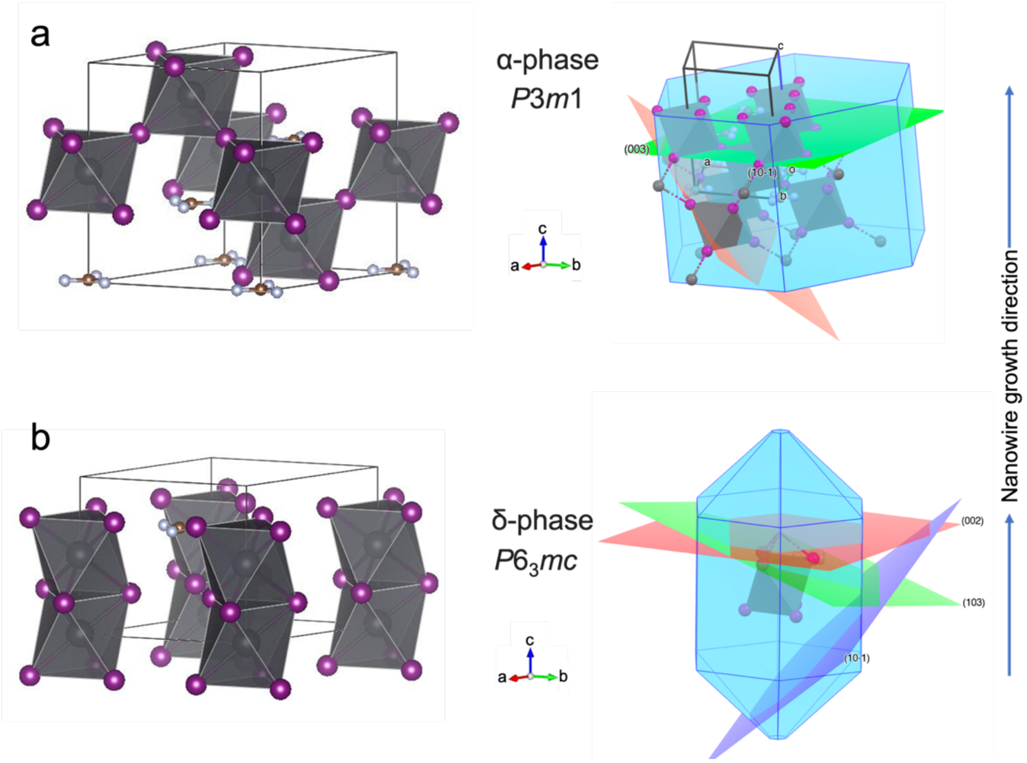

Figure S5. Crystal structure and simulated single crystal morphology (generated with Mercury software) of a) α-phase $FAPbI_3$ and b) δ-phase $FAPbI_3$.

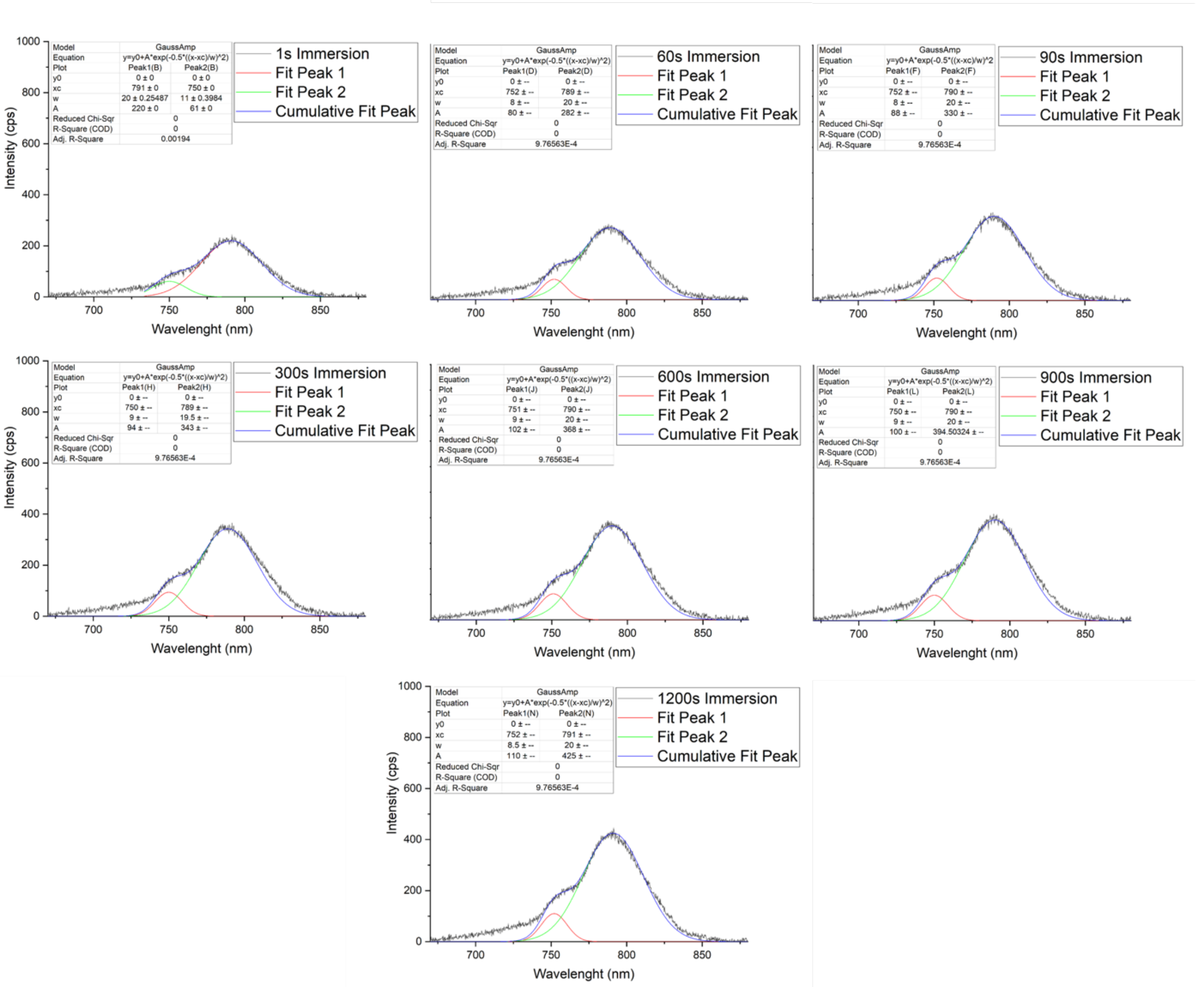

Figure S6. Gaussian curve fits of *in situ* PL spectra collected on PbI_2-infiltrated TiO_2 scaffolds at 1, 60, 90, 300, 600, 900 and 1200 s of immersion in FAI solution.

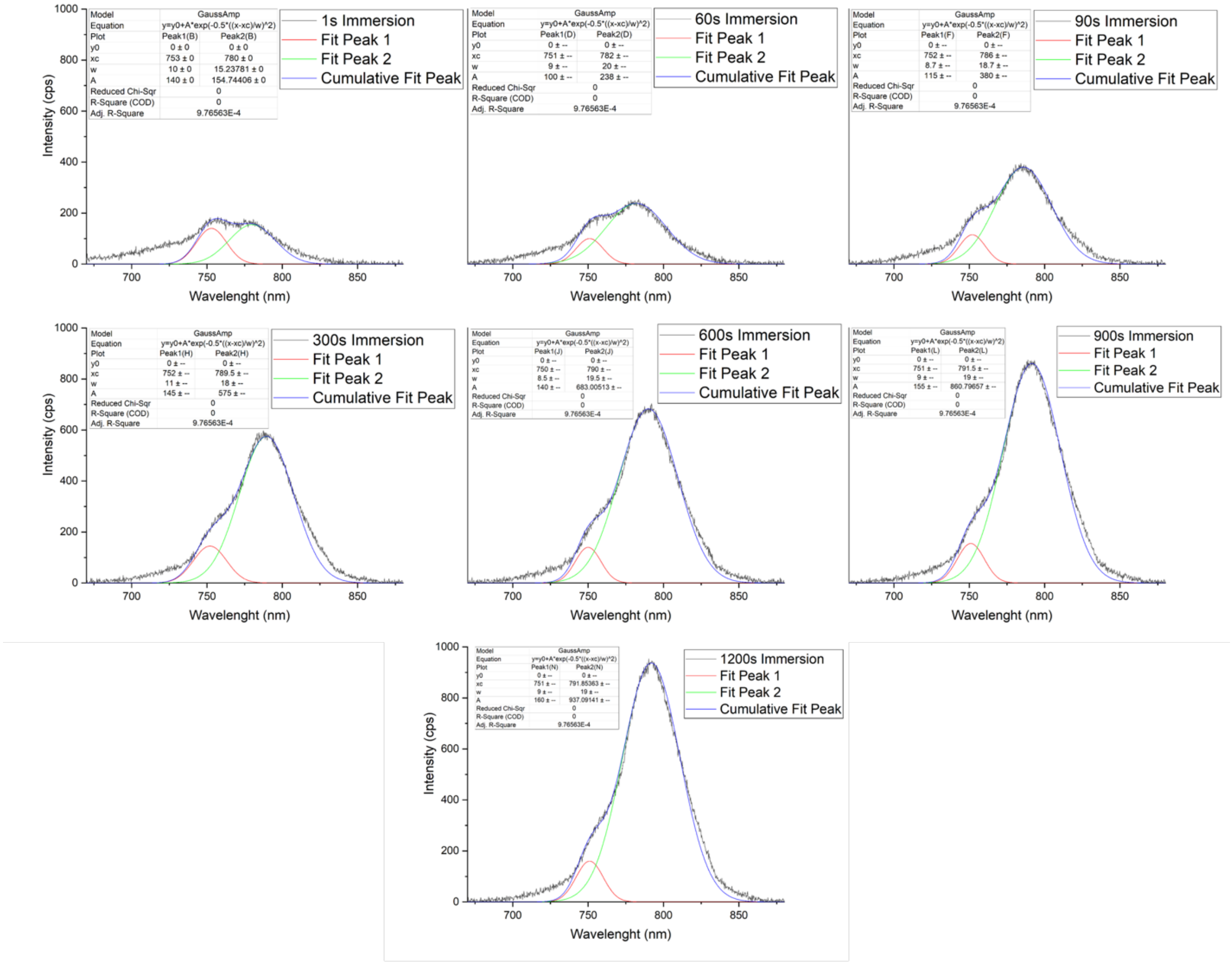

Figure S7. Gaussian curve fits for on *in situ* PL spectra collected on PbI_2-infiltrated TiO_2 scaffolds at 1, 60, 90, 300, 600, 900 and 1200 s of immersion in FAI/BAI solution.

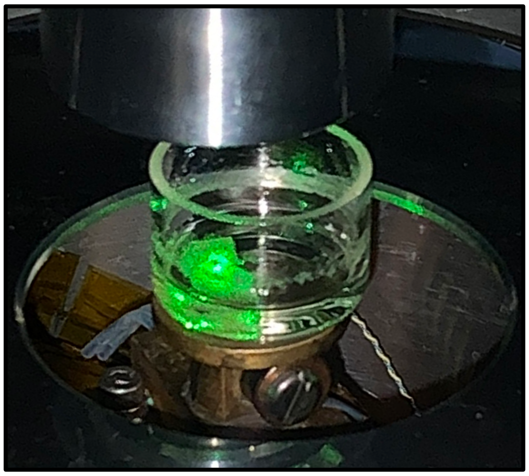

Figure S8. *In situ* PL measurement setup to collect spectra during immersion of PbI_2-infiltrated TiO_2 scaffolds in FAI or FAI/BAI solutions. λ_{ex} = 532 nm.

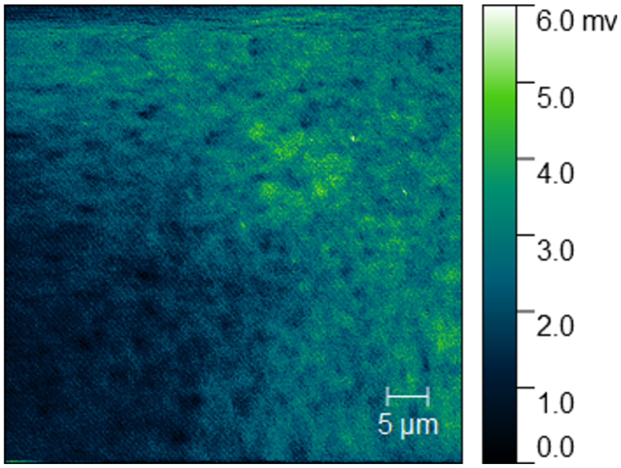

Figure S9. Hyperspectral PL map of a $FAPbI_3$ nanowire array formed by immersion of PbI_2-infiltrated anodized TiO_2 in a FAI solution for 600 s.

Table S1. Extracted parameters of double-exponential model fitted on TRPL spectra.

	τ_1	a_1	$\%a_1$	τ_2	a_2	$\%a_2$
$FAPbI_3$ thin film	5.23	0.79	63.2	63.95	0.46	36.8
$FAPbI_3$ nanowire	2.74	1.29	78.18	99.9	0.36	21.81
$FAPbI_3$ thin film+ HTM	1.78	3.31	93.5	63.33	0.23	6.49
$FAPbI_3$ nanowire+ HTM	2	3.08	92.77	35	0.24	7.22

.
